# Cholera

**Published:** 1849-03

**Authors:** 


THE WESTERN JOURNAL.

THIRD SERIES.—VOL. III.—NO. III.

LOUISVILLE, MARCH 1, 1849.

### CHOLERA.

This disease still lingers about New Orleans, and is extending slowly into other parts of the Valley of the Mississippi. As late as the middle of February deaths from it were occurring daily in Nashville and St. Louis, although it had not assumed an epidemic form, as we believe it will not while the weather continues cold. We refer our readers to the communication of Dr. Fenner for much valuable information concerning the epidemic as it appeared in New Orleans. He confirms the truth of the statement so often made, that cholera, in the forming stage, is almost always a curable disease, and that it rarely comes on without giving its subjects fair warning. In view of this well-established fact, it becomes a matter of the greatest importance that cities and towns should provide *Diarrhea hospitals*, to which the poor may apply for medicine and advice on the irruption of the epidemic, and that the people should be taught that *diarrhea* is *cholera* wherever the latter disease is prevailing.

We have seen letters from several New Orleans physicians in all of which it is stated that the premonitory diarrhea, in most cases, was effectually controlled by stimulants and anodynes, without the aid of any mercurial preparation. The fact is gratifying; we are always glad when we can find a disease manageable without mercury; but such is our confidence in its superiority over all other agents for restoring biliary secretion, that, we confess, we should not feel safe in dispensing with it in any case of diarrhea where cholera seemed to be impending. From what we have experienced in this disease, we should always advise calomel for the premonitory symptoms. The recumbent posture is also important, and hot applications to the skin, and internal stimulants favor the action of the liver. Quinine was found useful in the epidemic at New Orleans, as it was here also in one or two cases in which it was tried. Considering the close resemblance of cholera to malignant intermittent fever, quinine would seem to be the remedy for warding off the dreaded collapse. Few complaints are more tractable than congestive fever; few cases are more hopeless than a congestive chill. The safety of the patient lies in averting the collapse.

In regard to hygienic measures, the most valuable are temperance in all things, cheerfulness, cleanliness, and ventilation. It has been remarked how the poor have suffered from the epidemic everywhere. This is mainly owing to neglect, the disease being permitted to run into the incurable stage before resorting to medicine; but a part is due also to the character of their dwellings. Dr. Christison perhaps goes too far when he says, "cleanliness and ventilation will dissipate any epidemic;" but no one will deny that they are the chief prophylactics against cholera. Every source of impurity ought to be removed from about our houses. Special attention should be paid to the sleeping apartments. They ought to be dry, elevated as much as possible, and so arranged as to admit of a free circulation of air through them. Upper chambers, during epidemics, have proved to be more healthful than those near the ground, and hence, if cholera should become epidemic, servants and all persons sleeping in the basements of houses should be removed to upper stories. Crowding should be avoided, for it has been proved in a multitude of instances that an atmosphere is created about the persons of cholera patients congregated together in which the disease is easily propagated. The question is often asked, "shall I drink brandy to keep off cholera?" We answer, all sudden changes of habit are hazardous. If you are a brandy-drinker, continue to use it while the epidemic prevails, and then abandon the pernicious practice; if you are not accustomed to its use, then do not begin it. Use brandy just as you would any other medicine. It is a valuable remedy when the disorder is upon you, but possesses no power to avert an attack. Practice temperance, exercise cheerfulness, wait upon your friends and neighbors when overtaken by the disease without any fear of contagion, take medicine, or send for your family physician on the first appearance of diarrhea—those who pursue this course, we fully believe, have but little to fear from cholera.

